# Dynamics, a Powerful Component of Current and Future in Silico Approaches for Protein Design and Engineering

**DOI:** 10.3390/ijms21082713

**Published:** 2020-04-14

**Authors:** Bartłomiej Surpeta, Carlos Eduardo Sequeiros-Borja, Jan Brezovsky

**Affiliations:** 1 Laboratory of Biomolecular Interactions and Transport, Department of Gene Expression, Institute of Molecular Biology and Biotechnology, Faculty of Biology, Adam Mickiewicz University, Uniwersytetu Poznanskiego 6, 61-614 Poznan, Poland; bartlomiej.surpeta@amu.edu.pl (B.S.); carseq@amu.edu.pl (C.E.S.-B.); 2 International Institute of Molecular and Cell Biology in Warsaw, Ks Trojdena 4, 02-109 Warsaw, Poland

**Keywords:** protein dynamics, protein engineering, hotspot prediction, mutational analysis, computational design, ligand transport, ensemble-based approach, flexible backbone, de novo design, rational design

## Abstract

Computational prediction has become an indispensable aid in the processes of engineering and designing proteins for various biotechnological applications. With the tremendous progress in more powerful computer hardware and more efficient algorithms, some of in silico tools and methods have started to apply the more realistic description of proteins as their conformational ensembles, making protein dynamics an integral part of their prediction workflows. To help protein engineers to harness benefits of considering dynamics in their designs, we surveyed new tools developed for analyses of conformational ensembles in order to select engineering hotspots and design mutations. Next, we discussed the collective evolution towards more flexible protein design methods, including ensemble-based approaches, knowledge-assisted methods, and provable algorithms. Finally, we highlighted apparent challenges that current approaches are facing and provided our perspectives on their further development.

## 1. Introduction

Due to their unique structural and functional properties, proteins constitute an essential element of life as well as various branches of the emerging sustainable economy [1,2,3,4,5]. However, only a few proteins are natively equipped with functional parameters and sufficient stability that are required for their industrial and medical utilization. Hence, protein engineering methods gained popularity as an efficient way to deliver new protein variants with desirable properties for a diverse range of tasks [6,7]. Directed evolution and rational design represent the mainstream approaches introduced in the last decades to deliver enhanced protein variants [8]. In essence, the directed evolution enables the generation of rather extensive mutant libraries by randomly introducing mutations in gene-encoding proteins. Generated variants are then evaluated, focusing on the property of interest [9,10]. The rational design originally incorporated expert knowledge and models of proteins from X-ray crystallography to successfully design a handful of mutations enhancing protein stability, function or solubility [11,12,13]. With the advent of high-performance computing, rational design processes have progressively relied on computational analyses of these static structures [14,15,16]. So far, computational protein designs have managed to predict not only smart libraries of improved proteins but also massive modifications of proteins towards novel functions [17,18,19].

**Figure 1:**
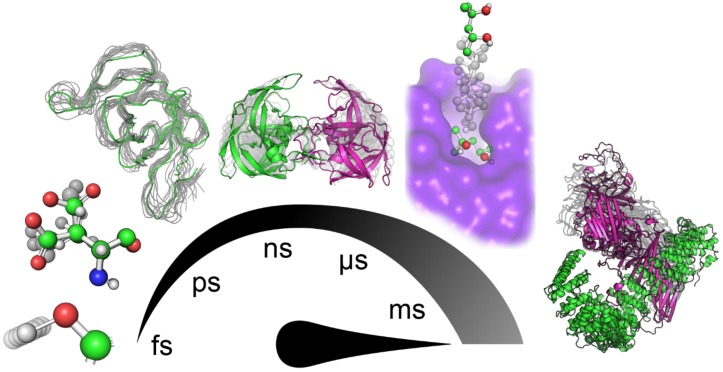
Hierarchy of principal motions in protein dynamics. From left to right: bond vibrations (fs–ps), side-chain rotations (ps–ns), backbone fluctuations (ns), loop motion/gating (ns–ms), ligand binding/unbinding events (>100 ns), and collective domain movement (>µs).

However, proteins are known to be dynamical entities, performing their function as an ensemble of diverse conformations rather than a single static structure. Protein dynamics is a highly complex phenomenon comprising numerous contributions from motions with different mechanisms of action and happening with diverse timescales and amplitudes (Figure 1) that highly depend on the system and the local environment [20,21]. Subangstrom vibrations of covalent bonds represent the fastest of those movements. The exploration of various rotamers of side-chains and fluctuations of the protein backbone involve nontrivial moves that span the space of several angstroms. In protein cores, such moves can require several nanoseconds to execute due to the necessity to synchronize with changes in surrounding residues [22,23,24]. Many conformational changes involve slower and more prominent coordinated movements of several residues in a sequence that manifests as, for example, gating movement executed by loops surrounding the active sites of many proteins [25]. In ligand binding and unbinding events, especially when the binding site is deeply buried in the protein structure, ligands often have to travel tens of angstroms. Such a transport process requires a series of systematic adjustments of protein side-chains and backbones along the traversed paths that might take up hundreds of milliseconds to occur [26]. Among the slowest principal motions performed by proteins are highly organized collective translocations of whole domains, starting on microsecond timescales and with amplitudes reaching nanometers. Finally, the most extensive conformational change transpires during the protein (un)folding processes, which can take hours and even days, and as such, is out of the scope of this review [22,23,24].

When we consider the reliable treatment of protein dynamics as an essential component of a successful protein design, it is natural to resort to the molecular dynamics (MD) simulation technique as a golden standard to investigate the conformational behavior of a protein. Nowadays, various MD simulation protocols can be utilized to deliver insights into protein dynamics on millisecond timescales with the growing utilization of graphics processing unit (GPU)-enabled parallelism and the development of more efficient software, gradually making such simulations even more affordable [27,28,29,30,31]. Despite all these improvements, MD simulations are not without errors in reproducing a realistic protein ensemble and, hence their experimental confirmation is necessary. Among the major limitations is the accuracy of force fields used to calculate interatomic interactions and the tractable sampling of the ensemble discussed above. The quality of traditionally applied force fields is intrinsically limited by numerous approximations like the lack of particular interaction types [32], neglect of electronic polarizability [33], and fixed protonation states of titrable residues [34]. At the expense of increased computational demands, some of those limitations can be partially overcome by improving potential models [35], resorting to polarizable force fields [36], and constant pH simulations [37]. Nonetheless, even without these advances, MD simulations relying on the latest force fields have been shown to reach chemical accuracy in their predictions for many different scenarios [38,39,40].

Regarding their utilization for protein engineering, MD simulations are commonly incorporated into different stages of the design process in order to modulate protein stability, alter interactions of proteins with cognate ligands or perturb dynamics of functional sites [41]. Next, the behavior of protein variants can be closely followed by MD simulations, allowing for the identification, ranking, and selection of promising candidates for experimental validation [42]. In recent years, efforts towards the possibility of also exploiting more distal positions during protein engineering have been gaining momentum [43,44,45,46,47]. By allosteric action, mutations at these positions often affect the preference of proteins to adopt a dominant conformational state, enabling the engineering of proteins with altered selectivity [48,49] or even adopting novel functions [50,51]. As showcased by those mentioned above and other studies [52,53,54,55,56,57], the crucial role of more comprehensive treatments of protein dynamics for the success of de novo designs, as well as the modification of existing proteins, is well recognized by now.

In this review, we focus on the recent developments in computational methods and tools, which aim to overcome significant challenges brought by integrating protein dynamics into predictions. First, we discuss tools developed for analyzing the fluid nature of interactions in protein ensembles and the elusive transport of ligands in a user-friendly way. In the second part, we critically review the efforts towards the efficient integration of protein flexibility on the backbone level into protein designs and engineering algorithms that are available in established software packages.

## 2. Tools to Facilitate Analyses of MD Simulation

Accessing information embedded in trajectories produced by MD simulations is a nontrivial task, especially when we focus on phenomena as complex as the networks of interacting residues and their correlated motions or as rare as the events connected with small molecules permeating through protein structures. To alleviate these challenges, we provide an overview of four recently developed tools aiming at understanding and controlling protein allostery and two tools that provide insights into the transport of small molecules (Table 1).

### 2.1. Interaction Network and Correlated Motion Analyses

**Figure 2:**
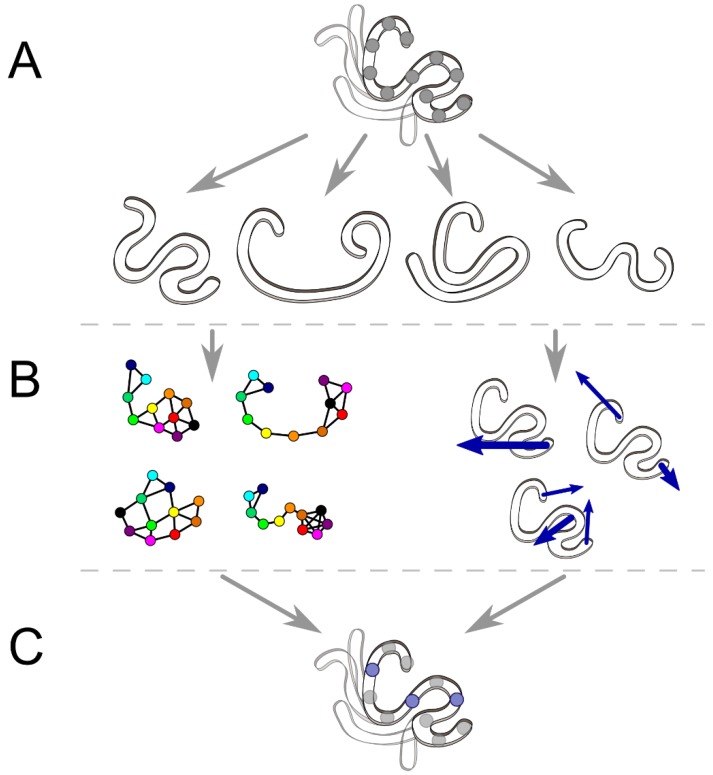
Predicting engineering hotspots for protein dynamics based on analyses of interaction networks and coordinated movements. (**A**) Functional protein dynamics can be represented by a conformational ensemble of a given protein. (**B**) This ensemble can be subjected to contact analysis to identify residue–residue interaction networks (left) or subjected to PCA to reveal coupled movements indicated by blue arrows right). (**C**) Either of these two approaches or their combination and hotspot residues (blue spheres) essential for the dynamics or allosteric communication can be selected for engineering.

Protein stability and function are dependent on their three-dimensional structures and are frequently conditioned by elaborate networks of noncovalent interactions between numerous residues [66]. Those networks undergo continuous dynamic changes by conformational rearrangement, which can be captured at atomic resolution using MD simulations [67,68,69] (Figure 2). Due to the inherent complexity in the detection and analysis of those changes, the simultaneous applications of several tools are frequently required. When enumerating a residue interaction network in an ensemble of protein structures from MD simulations, most of the available tools focus on coarse-grained networks consisting of Cα or Cβ atoms only [70,71]. To quantitatively explore the coordinated motions in the network, the use of principal component analysis (PCA)-based methods is considered an efficient strategy [72,73].

To provide a comprehensive view of interactions, the residue interaction network in protein molecular dynamics (RIP-MD) tool was developed [58]. RIP-MD can detect different nonbonded interactions including hydrogen bonds, salt bridges, van der Waals, cation–π, π–π, arginine–arginine, and Coulomb interactions. As an input, RIP-MD requires a static protein structure in a PDB format (web server) or MD trajectory in a DCD binary format (standalone and VMD plugin). The input is initially processed by removing heteroatoms, adding missing protein atoms and extracting parameters such as partial charges, Lennard-Jones parameters, secondary structure classification, and solvent accessibility. As an output, network files, including residue interaction networks for each interaction type and a combined network, are provided. The network files also store information about the secondary structure and the solvent accessibility. Furthermore, Pearson correlation plots are generated to detect possible behavior relationships between interacting residues. In a case study of the soluble myeloid differentiation-2 protein, RIP-MD was able to detect differences in interactions occurring in different conformational states, suggesting that the closing process increases the number of interactions and reduces the interaction correlations in the closed state. Further work is ongoing to broaden the capabilities of RIP-MD by accounting for interactions with nonprotein species [58]. This addition to the analysis will capture the effect of the environment and interactions with cognate ligands on proteins, which may be beneficial for protein engineering in particular.

A new software package, Java-based Essential Dynamics (JED), was developed to facilitate comparative PCAs of MD simulations of different proteins [59], including their apo- and holoforms, as well as wild-type and mutant variants. In the initial stage, the coarse-grained Cα atoms analysis of an ensemble, provided as PDB files, is performed to generate a pre-PCA output comprising a matrix of atomic coordinates, an overall root-mean-square deviation (RMSD), and an RMSD per residue. Then, the PCA of Cartesian-based coordinates, the PCA of internal distance pairs, or both analyses can be performed, optionally having less relevant modes and outlying PCA variables removed based on user-specified cutoffs. The output consists of files containing displacement vectors, covariance, correlation and partial correlation matrices, eigenvalues, and the most relevant principal components derived from the matrices. The analyses of both covariance and correlation are highly recommended, since they vary in the descriptions of collective motions concerning their amplitudes that are often sensitive to the mutation to a different degree. Finally, essential motions based on the matrices can be visualized, approximating the protein motions at various timescales. To compare dynamics among different proteins or different variants of the same protein, JED can compute cumulative overlaps, root-mean-square inner products, and principle angles. Depending on the degree of the overlaps in these features, the similarity in the protein dynamics can be established. As a case study, the authors analyzed 100 ns long simulations of a single-chain variable-fragment (scFv) antibody and its single-point mutant [59]. The detected disparities in correlation matrices, the PCA results, and the correlated residue pairs indicated that JED is sensitive enough to compare protein design evaluations [59].

Romero-Rivera and coworkers proposed a promising protocol combining information on residues proximity and their correlated movements into the so-called shortest path map (SPM), which can be applied to infer allosteric communication within a protein structure [43]. The first step in generating an SPM is the construction of a graph, in which Cα atoms of residues represent nodes and edges are drawn between pairs of nodes maintaining their distance below 5 Å in the whole MD trajectory. The edge lengths are then assigned based on the correlation coefficient between the connected Cα atoms in an inversed manner, i.e., larger coefficients result in shorter edges and vice versa. Next, the Dijkstra algorithm [74] is used to simplify the graph by identifying the shortest paths throughout the whole protein. Finally, pairs of residues that contribute the most to these paths are located representing central points for the communication. The SPM approach has been implemented in the DynaComm tool, and the development of a web server is ongoing [43]. By combining the SPM approach with PCA, the authors were able to identify the key positions that were previously mutated during the laboratory optimization of a computationally designed retro-aldolase by directed evolution [43]. This indicated rational design guided by SPM and PCA could help to identify distal mutations important for engineering of more efficient proteins akin to those produced by directed evolution experiments.

Similarly, by combining network analyses with PCA, the computation of allosteric mechanism by evaluating residue–residue associations (CAMERRA) tool aims to capture allosteric motions based on the residue–residue contact analysis of protein dynamics [60,61]. The CAMERRA tool is freely available as a set of Perl scripts. The required input for CAMERRA operation is an all-atom ensemble of diverse conformations of the investigated protein supplemented as a set of PDB files. In the beginning, residue–residue and residue–ligand contact matrices describing electrostatic, van der Waals, and hydrogen bond interactions are computed, resulting in contact matrices that are further condensed to form a mean contact matrix. Consequently, the mean contact matrix is exploited to generate a covariance matrix by computing the correlation between a pair of relevant contacts using a four-point correlation. Such an analysis may be able to capture crosstalk between the residues that lead to the formation or disruption of other contacts, therefore providing insight into the mechanisms of an allosteric network. Finally, a PCA is performed on the covariance matrix of the contacts, directly uncovering the displacement modes of the contacts (creations and disruptions), which is advantageous for understanding essential motions of biopolymers. This method was successfully applied to study several novel allosteric mechanisms including a frustrated fit mechanism and negative allostery in a retinoid X receptor complex [75] or the pressure activation of a lipase [76].

### 2.2. Analyses of Ligand Transport

Detailed tracking and analysis of ligand behavior across MD trajectories of biomolecular systems represent another strategy to enrich the protein design process by highlighting regions crucial for the transport of ligands, i.e., molecular tunnels, channels, and gates [25,77], which determine ligand associations and dissociation mechanisms [78,79]. In such a way, structural hotspot residues can be detected and considered during the protein engineering process to improve protein activity, change selectivity, or stability [80]. Readers interested in current approaches to simulations of ligand transport can refer to the recent expert review by Nunes-Alves and coworkers [81].

**Figure 3:**
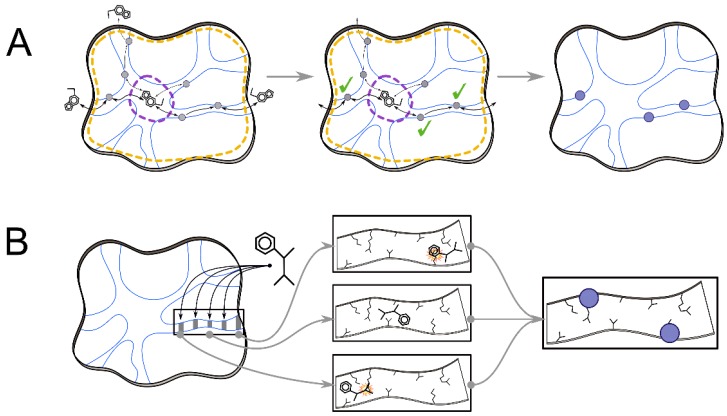
Hotspot detection based on ligand transport analyses. (**A**) AQUA-DUCT tool traces the movement of ligands via void spaces (blue lines) inside the scope region (dotted orange shapes) of the protein moiety throughout an MD trajectory. Only the ligands that reach the functionally important object region (dotted violet ellipses) are considered. The significance of the interactions of transported ligands with residues (grey spheres) along the ligand trajectory (black arrows) can be evaluated to select relevant hotspots (blue spheres) for the modification of the transport kinetics. (**B**) By iteratively docking the ligand along a molecular tunnel, CaverDock estimates the energy profile of a ligand transport, indicating residues that are most likely responsible for energy barriers in the path. These residues represent hotspots (blue spheres) for the design of new protein variants with altered ligand transport.

AQUA-DUCT [62,63] aims to provide detailed insights into the process of how a given type of molecules, such as water, ions, gasses, or any other kind of ligand, penetrates through the selected region of a protein (Figure 3A). As a minimal input, the user has to provide an MD trajectory and a configuration file describing two important regions for the analysis and defining the traced ligand. The first region is called a scope, which usually covers the whole protein. The second region is called an object and represents a functionally relevant region of interest, for example, the active site of an enzyme. An initial step in the workflow is to detect all traceable residues that reach the object and track their motions within the scope along the trajectory producing the so-called raw paths of ligands. Each path is then analyzed to identify possible repetitive events of a given ligand transiting between the object, scope, and surroundings, thereby dividing the raw paths to separate three types: (i) incoming path, (ii) outgoing path, and (iii) object for paths of ligands residing within the protein. In the following step, separate incoming and outgoing paths are assigned as inlets, i.e., paths connecting the exterior of the scope with the object region in any direction. Finally, the identified inlets are clustered, resulting in the pathways of the protein structure. Additionally, a statistical analysis is performed for all clusters, enumerating the number of the evaluated molecules, paths, inlets, and clusters, and several more specific statistics, including the lengths of the paths or the durations of the transport events. To illustrate the computational demands, the AQUA-DUCT analysis of 100 ns long MD simulations of murine epoxide hydrolase (4992 protein atoms) surrounded by 8488 water molecules requires 8–12 h to execute on a powerful workstation (Intel Core i7 CPU @ 3.50GHz machine, 64 GB RAM) [62]. For visualization purposes, a PyMOL [82] script or session can be generated according to user specifications. The presented method provides an efficient and robust way of detecting the usage of transport pathways in protein structures, including the detailed tracing of a specified ligand type, which is a challenging task, especially when considering thousands of water molecules in a trajectory composed of thousands of snapshots. In a follow-up study, the authors used MD simulations with AQUA-DUCT to examine the internal architecture of epoxide hydrolase from *Solanum tuberosum*, and based on their experience, they designed a relatively straightforward protocol for the detailed analysis of cavities networks and tunnels capable of pinpointing hotspots for engineering experiments [83]. Such an approach was integrated into the engineering workflows of Subramanian and coworkers on cupin-type phosphoglucose isomerase from *Pyrococcus furiosus* [84] and d-amino acid oxidase (DAAO) [85]. In these studies, the tracking of ligands and water molecules with AQUA-DUCT helped to detected important features related to transport phenomena and to identify remote mutations governing the specificity and activity of these enzymes [84,85].

As an alternative to very costly explicit MD simulations, the passage of ligands through biomolecules can be explored by docking these ligands to an ensemble of precomputed molecular tunnels with CaverDock software [64,65] (Figure 3B). Benefiting from the fast operation of CaverDock calculation, it is possible to run the calculations over such an ensemble for multiple different ligands. For CaverDock operation, tunnels must be represented as sequences of spheres for each given conformation of a macromolecule. Such input data can be easily generated by CAVER 3.0 software [86]. The input spheres of each tunnel are then discretized into a set of discs, which represent planar constrains for the subsequent placement of a ligand with the AutoDock Vina molecular docking tool [87]. Such an approach is, however, inherently noncontinuous, as some bottlenecks can be avoided by the ligand abruptly changing its orientation and/or conformation. A solution to generate a fully continuous trajectory adopted by CaverDock is to restrict conformational changes of the ligand during its transition from one disk to the next. Since the more advanced approach accentuates unrealistically high-energy barriers due to the rigid-protein docking approach, CaverDock can also utilize the flexible docking procedure available in AutoDock Vina. Such flexibility is capable of opening the narrowest sections of the investigated tunnels connected with the high-energy barriers, enabling the passage of various ligands via tunnels in cytochrome P450 17A1 and leukotriene A4 hydrolase/aminopeptidase [88]. Dealing with flexible residues during docking is more computationally demanding and should be used cautiously, as it can lead to the generation of the unrealistic conformation of flexible residues [65]. Marques et al. benchmarked the capabilities of CaverDock for protein engineering against predictions from sophisticated metadynamics, adaptive sampling, and funnel-metadynamics techniques [89]. In this detailed comparative study, the transport of ligands in two variants of haloalkane dehalogenase was investigated, and based on the analysis of energetic and structural bottlenecks, several residues playing a crucial role in the ligand-transport process were identified, some of them were previously mutated to engineer a very proficient biodegradator of a toxic anthropogenic pollutant 1,2,3-trichloropropane [90,91]. Overall, CaverDock reached good qualitative agreement with the rigorous MD simulations in this model system attesting its applicability for the engineering of ligand transport phenomena [89].

## 3. Advances in the Integration of Protein Flexibility into Protein Design and Redesign Methods

**Figure 4:**
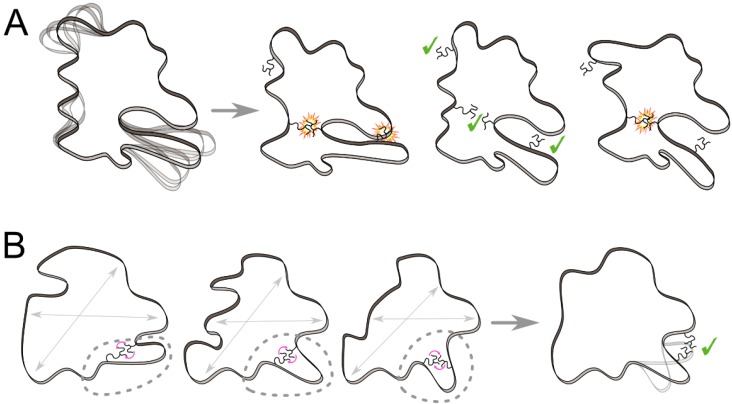
Flexible-backbone approaches facilitating the successful design of more diverse protein variants. (**A**) By employing a structural ensemble of a given protein, a larger variety of residues can be introduced to additional positions (green ticks), including those buried in the protein core, which would otherwise cause steric clashes (orange explosion-like shapes). (**B**) Data on protein dynamics encoded in different experimental structures or predicted ensembles can be extracted in the form of tertiary motifs (grey dotted circle) of interacting residues (pink arrows). Analogously, machine learning methods can learn and generalize the data to inspire novel backbone movements (grey arrows). The derived knowledge then enables the efficient application of more pronounced, yet physically correct, backbone perturbations during the design procedure.

During the past few years, we have witnessed a surge in the efforts to develop novel design methods capable of robust treatments of protein dynamics (Table 2). These methods can be divided into the following three categories: (i) methods utilizing pregenerated molecular ensembles (Section 3.1; Figure 4A), (ii) knowledge-based approaches to generating more pronounced backbone perturbations effectively (Section 3.2; Figure 4B), and (iii) provable design algorithms with extended backbone flexibility (Section 3.3).

### 3.1. Ensemble-Based Approaches

The generation of molecular ensembles by using MD and Monte Carlo (MC) simulations has become more affordable for a wider group of users, creating a means to face novel protein design challenges. By utilizing conformational ensembles, protein design algorithms can take the dynamic nature of the protein structures into account, providing a biologically sound strategy and frequently improving the performance of the employed methods [98,99].

We start this section by reviewing insights from two studies aiming at benchmarking generic procedures for ensemble generation on the success of protein design or redesign tasks. In the first comparative research by Ludwiczak and colleagues, 10 protocols combining methods from Rosetta software [100] with MD simulations were applied to 12 diverse proteins [54]. For protein redesign, three distinct structural ensembles were obtained using MD simulation, MD simulation followed by the introduction of small backbone perturbations with Rosetta Backrub [101], or Rosetta Backrub alone. Subsequently, the protein sequences were redesigned using either the fixed backbone (FixBB) or design-and-relax (D&R) methods on each ensemble [102,103]. We note here that the employed simulations were run for four ns, although with 50 replicas, representing somewhat limited sampling around the conformational minima even though the target proteins were relatively small (up to 103 residues). The designed sequences were analyzed based on entropy, covariation, profile similarity, and packing quality in the corresponding generated structures. The best performance was observed for the protocol using MD simulation in combination with Rosetta Backrub for the ensemble generation, followed by redesign with the D&R method. This time, analogous protocols were tested for de novo design purposes using only the more efficient D&R method, confirming that the procedure based on the MD simulation coupled with Rosetta Backrub yielded the best results. In the second benchmarking study, Loshbaugh and Kortemme performed a comprehensive evaluation of four different flexible backbone design methods available within the Rosetta software using six datasets [104]. Comparing FastDesign [105,106], Backrub Ensemble Design [107], CoupledMoves with Backrub [52], and CoupledMoves with kinematic closure, the authors concluded that the CoupledMoves method performs better in recapitulating sequences of known proteins compared to the other two alternatives. This finding highlights the importance of incorporating the side-chain and backbone flexibility simultaneously during the design. Interestingly, all methods performed poorly on two deep sequencing datasets, which should be taken with caution when applying Rosetta for such purposes. Overall, both studies emphasize that flexible backbone approaches combined with side-chain flexibility can significantly outperform methods utilizing only a single conformation.

The predictive performance of the Flex ddG method in estimating the change in binding free energy after mutation (ΔΔG) at protein–protein interfaces has also been boosted when using a structural ensemble instead of a single static structure [92]. In this method, an ensemble of up to 50 structures is generated by the conformational sampling in the surroundings of mutated sites with the Rosetta Backrub program. Then, the wild-type ensemble is optimized by repacking side-chains and performing energy minimization. To generate a mutant ensemble, the mutation of interest is introduced to each structure before conducting the analogous side-chain repacking and minimization. Finally, both ensembles are scored to calculate the ensemble-averaged ΔΔG. The method was validated using the ZEMu dataset of 1240 mutations [108] derived from the SKEMPI database [109]. For this dataset, the Flex ddG method reached a Pearson correlation coefficient (PCC) of 0.63 and an average absolute error of 0.96 Rosetta energy units. The enhanced performance was especially prominent in the case of small-to-large mutations, emphasizing that backbone flexibility constitutes a key factor during the modeling of these mutations. Relevant improvements were also achieved for modeling stabilizing mutations and mutating antibody–antigen interfaces. Interestingly, the enhanced performance over a fixed backbone approach was observed already when averaging over 20–30 conformations, a relatively low number in contrast to by previous ensemble-based methods, for which thousands of structural models were required [110].

Notably, the Flex ddG method was evaluated in three comprehensive benchmarking studies focusing on different engineering scenarios. Aldeghi and coworkers evaluated alchemical free-energy calculations and three Rosetta protocols including Flex ddG in combination with different force fields for the prediction of changes in binding the affinity of ligands upon mutation [111]. In total, 134 mutations were considered for 27 ligands and 17 proteins, showing that Flex ddG can reach quantitative agreement with such experimental data with a root-mean-square error (RMSE) of 1.46 kcal/mol and a PCC of 0.25, which was on par with the best performing alchemical calculations (an RMSE of 1.39 kcal/mol and a PCC of 0.43) [111]. At this point, it is worth comparing the computational resources required for such predictions. The alchemical calculations were reported to take two to five days using 20 CPU threads and one GPU, while Flex ddG computations were usually finished within a day on a single CPU core [111]. The same author collective also evaluated the utilization of these methods for the prediction of 31 drug resistance-conferring mutations for eight tyrosine kinase inhibitors of human kinase ABL [112]. For this dataset, Flex ddG was found to be highly accurate with an RMSE of 0.72 kcal/mol and a PCC of 0.67, even outperforming the much more demanding alchemical calculations [112]. Interestingly, significant improvements in ΔΔG prediction could be reached with a consensus of predictions from Flex ddG and alchemical calculations in both studies [111,112]. Another comparative study investigated the performance of five predictive tools when applied for alanine scanning to identify hotspot residues at protein–protein interfaces [113]. For a dataset of 748 single-point mutations to alanine from the SKEMPI database, Flex ddG ranked the best (PCC of 0.51) from the tools that were not trained using this database [113].

The advantages of incorporating conformational ensembles during design have also been noted during the development of a multistate framework that enables the adoption of reliable methods implemented in the Rosetta package for single-state design (SSD) and also for multistate design (MSD) [93]. Briefly explaining the mode of action, the input for the framework consists of a set of multiple states (structural conformations) and the population of sequences generated by randomly introduced single-point mutations, which are processed and altered by a genetic algorithm. Next, each sequence–state pair is evaluated and scored based on the Rosetta SSD protocol of the user’s choice. The score of each sequence are communicated back to a sequence optimizer to perform the next iteration, until the fitness criteria are satisfied, finally giving a population of the optimized sequences. This is opposite to the standard SSD, which uses an MC algorithm and produces only a single sequence. The performance of MSD was evaluated on several design perspectives. Firstly, the performances of MSD and SSD in the task of recapitulating the binding site in the human intestinal fatty acid-binding protein was compared utilizing its ensemble obtained by NMR spectroscopy. Here, the SSD approach was used separately for each conformation, while the MSD was run on the whole ensemble at once. The MSD procedure achieved higher average native sequence recovery (NSR) and native sequence similarity recovery (NSSR) rates. Additionally, de novo ligand-binding design was performed for 16 proteins using SSD and MSD, where conformational ensembles of 20 and 1000 structures were generated by the Rosetta Backrub algorithm and a 10 ns long MD simulation, respectively. In this comparison, the MSD approach primarily produced sequences with higher NSR and NSSR rates and slightly lower energies, proving the advantages of the ensemble utilization. Interestingly, the quality of the designs originating from Rosetta Backrub and MD simulations were comparable, even though the mean Cα RMSDs over the ensembles differed notably, which were 0.17 and 0.62 Å, respectively. Finally, the multistate framework was tested by introducing retro-aldolase activity into protein scaffolds, which revealed nine proteins with experimentally confirmed activities [93].

A similar idea of combining an ensemble-based design and a multistate approach was behind the development of a meta-multistate design procedure (meta-MSD) to rationally design proteins that spontaneously switch between conformational states [94]. In this case, the procedure started with the generation of an ensemble of backbone templates with Rosetta Backrub and PertMin approaches [99,114] to cover the conformational landscape, including all transition states of interest. Next, the whole ensemble was split into microstates that were energy-minimized. Then, these microstates were assigned to major, transition, and minor states based on their structural features. Finally, the sequences expected to transit between the states were identified based on their relative energies. Based on meta-MSD, several Streptococcal protein G domain β1 variants were engineered to obtain structures that can exchange conformations between two states spontaneously, producing experimentally validated protein exchangers capable of switching between the states on a millisecond timescale [94], thereby highlighting the importance of the accurate modeling of a local energy landscape for designing protein dynamics.

### 3.2. Knowledge-Based Approaches

Following the expansion of protein structure databases, which contain a considerable amount of data related to structure–dynamics–function relationships in proteins, new methods to assess backbone flexibility have been designed, benefiting from this wealth of knowledge. The methods introduced here are implemented in the Rosetta software and represent an exciting direction for improving protein design processes by more efficiently exploring alternative backbone conformations.

The first among the reviewed data-driven approaches is the flexible backbone learning by Gaussian processes (FlexiBaL-GP) method [95] that uses multiple structures of a given protein to learn the most probable global backbone movements specific for training structures using the Gaussian process latent variable model as a machine learning method. These learned movements are then applied to guide the search for proteins with alternative backbone conformations by Markov Chain Monte Carlo sampling, where at each step 95% of the time is spent on the selection of the optimal side-chain rotamers and 5% of the time is spent on the generation of the protein backbone movements. FlexiBaL-GP can utilize various sources of training data including X-ray structures, NMR models, and MD simulations. When learning from a set of 28 crystal structures of ubiquitin and using two latent variables, the FlexiBal-GP method generated an ensemble of structures for native ubiquitin with an RMSD range of 0.5–0.65 Å from a reference structure. Notably, the ensemble recovered over 40% of the conformational diversity of the ensemble obtained by NMR spectroscopy. Moreover, the method’s ability to enrich a library of ubiquitin variants towards those with improved affinity to ubiquitin carboxyl-terminal hydrolase 21 was evaluated. For this task, the FlexiBal-GP method was trained on two wild-type complexes only or combined with either a structure of a tightly binding mutant or MD-based ensembles starting from the two wild-type structures. All three derived models outperformed flexible designs with Rosetta Backrub, as well as designs based on ensembles generated with MD simulations and the constraint-based method, CONCOORD [115].

A different approach to harnessing knowledge from structural databases and to navigating sequence space sampling with a flexible backbone has been explored by the structural homology algorithm for protein design (SHADES) [96]. This approach relies on the libraries of In-contact amino acid residue TErtiary Motifs (ITEMs) derived from curated protein structures, in which local contacts were analyzed for each residue. Analogously, target ITEMs are then identified for each position in the target structure in a position-specific manner and matched to the ITEMs database in order to generate candidate ITEMs libraries. Finally, these libraries are exploited by an iterative population-based optimization method that substitutes all residues in each target ITEM position with all residues from a candidate ITEM. The structure of the altered fragment is then adjusted by optimizing its backbone with the Rosetta Backrub method, repacking the side-chains and minimizing or relaxing the whole structure with or without backbone restraints. Using a dataset of 40 proteins from different families, the SHADES performance in recovering the native sequences of the proteins was evaluated, reaching a 30% average sequence recovery and a 46% sequence similarity between the designed and natural proteins, when candidate ITEMs derived from homologous proteins were excluded. When the homologs were retained in the candidate libraries, the sequence recovery rate increased up to 93%. Notably, rather large conformational diversity was observed for the successfully designed models, in some instances exhibiting more than a 1 Å RMSD from their respective native structures. Overall, these tests indicated that SHADES could capture sequence–dynamics–structure relationships correctly while spending about 25 times less CPU time than the redesign mode of the Rosetta FastRelax method [116].

### 3.3. Provable Algorithms

Due to the high complexity of protein design tasks, especially when employing ensemble-based approaches (Section 3.1), the majority of the tools rely on heuristic algorithms as an expedient way to obtain the desired constructs. For more complicated tasks, these approaches are often barred from generating optimal solutions, which in turn can lead to the design of sequences that are not guaranteed to have the lowest energy [117]. In response to those limitations, provable algorithms have been developed, creating a promising alternative for reaching entrenched solutions [117,118]. Here, we briefly outline some of the most compelling developments that led to an advanced description of backbone flexibility. For a more comprehensive overview of provable algorithms and their evolution and application, please see the very insightful reviews published recently [119,120].

The development of provable algorithms started with the adaptation of the dead-end elimination (DEE) method [121] that was later improved by introducing rotamers’ minimization before pruning to enable a more continuous description of side-chains, an essential component of several successful designs [118,122]. The initial approach to backbone flexibility was introduced with the dead-end elimination with perturbations (DEEPer) method [123], relying on a predefined set of small local movements extracted from an experimental structure such as Backrub [124] or sheer. However, such motions are mostly restricted to subangstrom dimensions to avoid disruptive changes propagated to a distant region from the segment of the altered backbone. To enable more progressive motions in a predefined contiguous part of the backbone such as the movement of a flexible loop, the coordinates of atoms by Taylor series (CATS) approach was recently introduced [97]. The main idea of the approach lies in the new definition of the backbone internal coordinate system, which enables physically sensible, continuous, and strictly localized perturbations of the given segment of the backbone in a manner that is compatible with the advanced DEE workflows. The CATS method was tested on 28 different proteins with flexible backbone treatment enabled for five to nine-residue long segments. By introducing more pronounced changes in backbone conformations, almost 0.2 Å on average, CATS reached a mean improvement in design energies of 3.5 kcal/mol in comparison to the rigid-backbone approximation. Such an improvement is nearly twice as large as what was observed previously for restricted backbone perturbations introduced by the DEEPer method on the same set.

Owing to persistent optimization efforts [125,126,127,128], provable algorithms can nowadays be applied for protein design while simultaneously employing both the continuous flexibility of side-chains and enhanced backbone flexibility efficiently at similar computational costs to more rigid approaches. These methods are available in OSPREY 3.0 [129], in which the analysis speed has been further promoted by the newly supported use of GPUs and multicore CPUs for some of the modeling tasks, which were prohibitively complicated for the previous version of the software. As underlined by several studies featuring various applications of provable algorithms [130,131,132,133], these algorithms have matured enough to be of practical utility for protein engineers. This trend will undoubtedly gain further momentum with the recent developments discussed herein, even though their computational demands might still be limiting for some applications.

## 4. Conclusions, Challenges, and Perspectives

In contrast with proteins evolved through directed evolution, constructs predicted by computational protein engineering methods have so far been focusing mainly on hotspot residues close to functional sites. By considering the proximity of relevant regions, mutations have the highest chance of altering the target function, and at the same time, the number of variants to evaluate is kept tractable. Unfortunately, this restriction often hampers the performance of rationally designed proteins. It is clear that we need more efficient workflows and tools that can pinpoint hotspots at crucial distal sites as well. One class of such hotspots involves residues forming allosteric networks capable of inducing a shift in populations of protein conformations to support their altered function upon mutation. Here, we would like to highlight the availability of tools for rapid analyses of protein allostery focusing on residue–residue interactions in a single static structure or employing normal mode analysis (NMA) to approximate protein dynamics [134]. However, the performances of these approximate tools are often impeded by two factors: (i) the quality of a single-input structure and the extent, to which this structure represents essential interactions present in the conformational ensemble, and (ii) the limited sensitivity of underlying NMA to mutations that do not produce substantial conformational changes [135]. Those limitations are inherently overcome by ensemble-based approaches, in which network analyses of MD simulations are facilitated by the tools discussed in Section 2.1. The second class of remote hotspots is connected with ligand transport, a phenomenon that is hard to tackle due to its rare nature, which in turn requires extensive sampling. Currently, there are tools suitable for robust analyses of transport events captured by MD simulations and tools capable of the efficient exploration of a precomputed ensemble of transport tunnels in proteins by multiple ligands (Section 2.2). However, there is still a gap to close, before we can rationally design mutations enhancing ligand transport. In particular, effective means to predict how the ligand presence alters the dynamics of transport pathways to factor in ligand-specific effects of mutations [136] still have to be developed together with more efficient methods to sample the passage of ligands through structural ensembles of proteins.

Throughout this review, we have witnessed a consistent success of methods incorporating different degrees of protein dynamics in increasing the accuracy of their predictions owing to the innate ensemble nature of the proteins. These methods frequently require user expertise in complicated computational methods and protocols. Considering that some of fully automated and easy-to-use methods available nowadays originate from very sophisticated and computationally extensive approaches [137,138,139,140,141] and the ongoing rapid development of powerful technologies, in synergy with research on more efficient algorithms, we perceive recent advanced methods and algorithms reviewed here as heralded future automated methods accessible not only to specialists but also to researchers with much broader expertise. As various flexible backbone approaches will, due to their upcoming maturity and indisputable benefits, be gradually joining the mainstream protein design methods, the involvement of dynamics in engineering processes is likely to reveal new challenges to overcome.

First, the successful utilization of molecular ensembles in protein design and redesign is dependent on the quality of input ensembles emphasizing the importance of sufficient and representative sampling. Since this is not a trivial task, but rather an art itself, the ensemble-based approaches reviewed here employ limited sampling. Despite sampling somewhat restricts conformational changes in protein backbones, these approaches achieve substantial advantages over the predictions relying on a single structure. The systematic utilization of a more extensive sampling via much longer, enhanced, or adaptive simulations will be required to thoroughly describe more global conformational transitions [27,28,29,30,31]. Alternatively, with further expansion of the PDB database, the knowledge-based methods similar to those reviewed in Section 3.2 might be trained from data on particular proteins and families, hence providing more global, yet robust, moves compatible with a given fold to be considered during the design. Additionally, there is still largely unexplored potential to derive such system-specific moves from extensive MD simulations that have been shown to recapitulate the conformational behavior of many structured proteins [40,142].

Second, with the increasing amplitude of introduced perturbations, the protein structures will more frequently be drawn from the conformational space further away from the structures produced by protein crystallography. Following the precedent of unsatisfactory performance observed for simulations of intrinsically disordered proteins using standard force fields, which were developed for folded and stable protein structures [143,144], to what degree all energy terms of currently employed scoring functions will be applicable for the ranking of very flexible designs remains to be seen. In parallel, it is evident that the flexible-backbone approaches are more successful in introducing the bulkier and often more hydrophobic residues. This success, however, accentuates a well-known tendency of design methods to improve hydrophobic packing but not polar interaction networks, since hydrophobic interactions are more straightforward to sample than directional polar ones [145], which regularly results in the problematic solubility of the design proteins. To help to reverse this trend, the utilization of methods for the efficient prediction of hydrogen bond networks, akin to the recently developed MC HBNet protocol [146], would be required, especially when coupled with more continuous descriptions of side-chains to increase the number of accessible solutions.

## Figures and Tables

**Table 1:** Computational tools to extract valuable information for protein engineering from molecular dynamics (MD) simulations.

Tool	Target Property	Availability		Code	Core Method(s)	Input		Link	Reference
		Web Server	Standalone			Structure	Trajectory		
Residue interaction network in protein molecular dynamics (RIP-MD)	Interaction network	+	+	Python	Residue interaction network	+	+	http://dlab.cl/ripmd/	[58]
Java-based Essential Dynamics (JED)	Essential dynamics	-	+	Java	Principal component analysis (PCA)	-	+	https://github.com/charlesdavid/JED	[59]
DynaComm	Allostery	-	+	Python	Distance and correlation-based graphs, Dijkstra algorithm	+	+	https://silviaosuna.wordpress.com/tools/	[43]
Computation of allosteric mechanism by evaluating residue–residue associations (CAMERRA)	Allostery	-	+	Perl, Python, C	PCA, contact analysis	-	+	shenlab.utk.edu/camerra.html	[60,61]
AQUA-DUCT	Ligand movement	-	+	Python	Geometry analysis	-	+	www.aquaduct.pl	[62,63]
CaverDock	Ligand movement	+	+	Python	Molecular docking	+	+	https://loschmidt.chemi.muni.cz/caverdock/	[64,65]

**Table 2:** Computational protocols implementing protein flexibility for protein design and redesign.

Primary Package	Category	Method	Short Description	Input	Sampling of Side-Chain and Backbone Flexibility	Package	Add-Ons	Reference
Rosetta	Ensemble-based	Flex ddG	Estimating interface ∆∆G values upon mutation	Static structure	Backrub, torsion minimization, side-chain repacking	https://www.rosettacommons.org/software/	https://github.com/Kortemme-Lab/flex_ddG_tutorial	[92]
		Rosetta:MSF	Multistate framework using single-state protocols	Ensemble	Genetic algorithm based sequence optimizer and user-defined evaluator from Rosetta protocols	https://www.rosettacommons.org/software/	-	[93]
		Meta-multistate design (meta-MSD)	Engineering protein dynamics by meta-multistate design	Set of ensembles	Fast and accurate side-chain topology and energy refinement algorithm for sequence optimization; backbone-dependent rotamer library optimization for side-chains	https://www.rosettacommons.org/software/	PHOENIX scripts upon request	[94]
	Knowledge-based	Flexible backbone learning by Gaussian processes (FlexiBaL-GP)	Learning global protein backbone movements from multiple structures	Ensemble	Markov Chain Monte Carlo sampling—95% time spent on the side-chain selection and 5% time spent on the generation of the backbone movement	https://www.rosettacommons.org/software/	-	[95]
		Structural homology algorithm for protein design (SHADES)	Protein design guided by local structural environments from known structures	Static structure	Sequence assembly from fragments followed by backbone optimization, side-chains repacking, and structure relaxation	https://www.rosettacommons.org/software/	https://bitbucket.org/satsumaimo/shades/src/master/	[96]
OSPREY 3.0	Provable	Coordinates of atoms by Taylor series (CATS)	Enabling progressive backbone motions during protein design	Static structure	Continuous, strictly localized perturbations of the given segment of the backbone using a new internal coordinate system compatible with dead-end elimination workflows	https://github.com/donaldlab/OSPREY3	-	[97]

## Abbreviations

MD	molecular dynamics
GPU	graphics processing unit
PCA	principal component analysis
RIP-MD	residue interaction network in protein molecular dynamics
VMD	visual molecular dynamics
JED	Java-based Essential Dynamics
scFv	single-chain variable-fragment
PDB	protein data bank
RMSD	root-mean-square deviation
SPM	shortest path map
CAMERRA	computation of allosteric mechanism by evaluating residue–residue associations
DAAO	D-amino acid oxidase
MC	Monte Carlo
FixBB	fixed backbone
D&R	Design-and-relax
PCC	Pearson correlation coefficient
ΔΔG	change in binding free energy
RMSE	root-mean-square error
SSD	single-state design
MSD	multistate design
NMR	nuclear magnetic resonance
NSR	native sequence recovery
NSSR	native sequence similarity recovery
FlexiBaL-GP	flexible backbone learning by Gaussian processes
SHADES	structural homology algorithm for protein design
ITEM	In-contact amino acid residue tertiary motif
DEE	dead-end elimination
DEEPer	dead-end elimination with perturbations
CATS	coordinates of atoms by Taylor series
NMA	normal mode analysis
